# A Strategy for Genome-Wide Identification of Gene Based Polymorphisms in Rice Reveals Non-Synonymous Variation and Functional Genotypic Markers

**DOI:** 10.1371/journal.pone.0105335

**Published:** 2014-09-19

**Authors:** Subodh K. Srivastava, Pawel Wolinski, Andy Pereira

**Affiliations:** 1 Crop, Soil and Environmental Sciences, University of Arkansas, Fayetteville, Arkansas, United States of America; 2 Arkansas High Performance Computing Center, University of Arkansas, Fayetteville, Arkansas, United States of America; International Rice Research Institute, Philippines

## Abstract

The genetic diversity of plants has traditionally been employed to improve crop plants to suit human needs, and in the future feed the increasing population and protect crops from environmental stresses and climate change. Genome-wide sequencing is a reality and can be used to make association to crop traits to be utilized by high-throughput marker based selection methods. This study describes a strategy of using next generation sequencing (NGS) data from the rice genome to make comparisons to the high-quality reference genome, identify functional polymorphisms within genes that might result in function changes and be used to study correlations to traits and employed in genetic mapping. We analyzed the NGS data of Oryza sativa ssp indica cv. G4 covering 241 Mb with ∼20X coverage and compared to the reference genome of Oryza sativa ssp. japonica to describe the genome-wide distribution of gene-based single nucleotide polymorphisms (SNPs). The analysis shows that the 63% covered genome consists of 1.6 million SNPs with 6.9 SNPs/Kb, and including 80,146 insertions and 92,655 deletions (INDELs) genome-wide. There are a total of 1,139,801 intergenic SNPs, 295,136 SNPs in intronic/non-coding regions, 195,098 in coding regions, 23,242 SNPs at the five-prime (5′) UTR regions and 22,686 SNPs at the three-prime (3′) UTR region. SNP variation was found in 40,761 gene loci, which include 75,262 synonymous and 119,836 non-synonymous changes, and functional reading frame changes through 3,886 inducing STOP-codon (isSNP) and 729 preventing STOP-codon (psSNP) variation. There are quickly evolving 194 high SNP hotspot genes (>100 SNPs/gene), and 1,513 out of 2,458 transcription factors displaying 2,294 non-synonymous SNPs that can be a major source of phenotypic diversity within the species. All data is searchable at https://plantstress-pereira.uark.edu/oryza2. We envision that this strategy will be useful for the identification of genes for crop traits and molecular breeding of rice cultivars.

## Introduction

The world's geometrically growing population has passed 7 billion, and it is estimated that rice production must increase 24% by 2025 to meet the global population demand [1]. The rice genome, considered as a global heritage, has the information and resources to continue to feed half the population of the world. The diversity of rice across the world is an excellent resource to understand the genetic events of human selection for traits of utility, and to discover new alleles for traits using plant genomics as it represents a modest size of 389 Mb for a cereal genome [2]. The genomes of individuals from the same species vary in sequence as a result of the evolutionary process and contribute to some of the specific features in individual genotypes. Therefore, sequence polymorphisms have become a prime target of interest following genome sequencing projects, with analysis and utilization of the identified variation the next goal. Single nucleotide polymorphisms (SNPs) are increasingly becoming the marker system of choice and their analyses are a part of almost all aspects of applications of genomics.

SNP discovery is valuable for crop improvement in two fundamental ways. First, it reveals DNA variation among varieties, thus providing the tools for selection in breeding programs [3]. Secondly, it provides the highest level of accuracy in mapping or anchoring of all forms of the phenotype, including biochemical, metabolic, physiological, and phenotypic performance [4]. However, for many crop plants there are an exceptionally low number of validated SNP markers available, although they are needed in large numbers for studies on genetic variation, linkage mapping, population structure analysis, association studies, map-based gene isolation and plant breeding purposes [5]. Therefore, correct identification of SNPs and their potential effects or functional annotation are important for crop improvement that will enable the selection of useful alleles of candidate genes in designing breeding experiments. Genome-wide comprehensive identification of polymorphisms among individuals within a species is crucial to studying the genetic basis of phenotypic differences and for elucidating the evolutionary history of the species [6]. Large-scale polymorphism surveys have been reported for human [7], mouse [8] and *Arabidopsis thaliana* [9]. Natural phenotypic differences are now amenable to genetic dissection, right up to the identification of causal DNA polymorphisms and functional nucleotide polymorphisms accounting for plant intraspecific developmental diversity. These variations often affect gene structure and may contribute to interspecific phenotypic traits and adaptation [10]. The high quality sequence of rice genome *Oryza sativa* ssp. *japonica* cv. Nipponbare (*Os japonica*) and *Oryza sativa* ssp. *indica* cv. 9311 (*Os indica*) contains a wealth of information that can explain the large amount of morphological, physiological and ecological variation observed in many varieties cultivated for food, since there is a high level of variability present in diverged rice genotypes [11]–[13]. The rice genome has also been surveyed for polymorphisms to develop several alternative models that can explain contemporary patterns of polymorphism in rice, and establish the introgression patterns of shared SNPs that reveal the breeding history and relationships among the varieties [1], [14]. Rice has been extensively analyzed for SNP variation within the species, and to the reference genome for various purposes [12], including many studies related to genome-wide SNP analysis between the two model subspecies of rice. Some genotypes have been analyzed by deep sequencing, focused on non-synonymous coding SNPs (nsSNPs), and very few have been evaluated extensively for potential functions. At present, genome-wide gene based SNP analysis is still lacking, results of which could directly be utilized in crop improvement [1], [12], [15]–[17].

Rapid process in genomic information at whole genome sequence (WGS) level allow us to evaluate and analyze base by base structural variations for their similarity, and the differences reveal potential functional information. In recent years, natural variation has gained significant interest at the genome-wide level and the correlation of these variations to their agronomic traits has been the prime focus [14], [18]. Development of functional markers for candidate genes has been utilized in many crops including rice. The progress of new technologies enables us to analyze multiple high quality genomes. The availability of the high-quality rice reference genome and Next Generation Sequence (NGS) information for some of the important genotypes allow us to examine the tremendous diversity of the rice gene pool at a very fine scale [19], [20]. The analysis shown here is basically focused on genome-wide analysis of rice sequences to identify gene based non-synonymous SNPs at different levels of comparison to present a group of SNPs responsible for or that might play an important role at the functional level. Genome-wide SNP identification and correlation with agronomic traits could be helpful for trait characterization [15], [20]. We believe that although significant analysis has been carried out for identification of SNPs between these species, there is still missing functional information of identified SNPs. The present study will focus on two rice sub-species cultivars, Guangluai-4 and Nipponbare, of the two sequenced genomes *Oryza sativa* ssp. *indica* (*Os indica*) and *Oryza sativa* ssp. *japonica* (*Os japonica*) respectively for identification and functional annotation of SNPs between them. This analysis can lead to the development of functional markers focusing on gene based analysis for trait based SNP analysis and utilization for crop improvement.

Next generation sequencing technology coupled with the growing number of genome sequences, more particularly from within the species, open the opportunities to redesign genotyping strategies based on focused genic information for more effective genetic mapping and genome analysis. Recently, genome-wide variation patterns in rice have been obtained from millions of high-quality SNPs and identified thousands of genes with significantly lower diversity in cultivated compared to wild rice, and a few of these variants have been found associated with important biological features [8], [20]. The development of NGS and analysis technology empower us to analyze and decode specific information in form of genetic variation (SNPs, InDels). The occurrence and virtual identification of enormous numbers of differences in individual nucleotides between individuals, enables every SNP to be a potentially useful marker [17]. Recently, sequence-based polymorphisms have been given much attention to explore the gene-based variation between high quality genomes and their related genotypes [15], [21]. This sequence based marker technology allows the development of molecular markers to target genomic regions of the sequenced genome using the NGS which facilitates isolation of co-dominants molecular markers for the targeted genomics region of any animal and plant species. This will also particularly facilitate the development of high-density molecular maps, essential for gene based cloning using genetic map positions, and identification of linked molecular markers for selecting desirable genotype in plant breeding programs.

DNA polymorphisms have been identified both in animals and plants. In animals, more than 300 diseases and traits have been studied using GWASs [22]. Among plants, in rice over 600 genes have been cloned using various functional genomics methods. Many of these genes control agriculturally useful traits such as yield, grain quality, resistances to biotic and abiotic stresses and nutrient use efficiency thus have potential utility in crop improvement [23]–[25]. For example, an SNP in the 5′ regulatory region of the *qSH1* gene was shown to cause loss of seed shattering owing to the absence of an abscission layer formation in rice. Another example is the *GIF1* (*grain incomplete filling 1*) gene that shows a restricted expression pattern during grain-filling compared to the wild rice allele, is probably a result of accumulated mutation in the gene's regulatory sequence through domestication [17]. Many of the gene specific SNPs can be important for trait variation, which led us to conduct genome-wide analysis of gene based SNPs to decode the genic variation underlying functions.

## Materials and Methods

### Data retrieval and analysis

The 73 bp reads of FASTQ data of *O. sativa* ssp. *indica* cv. Guangluai-4 (*Os indica*) with 20× genome coverage by next generation sequencing (SRA study: ERP000235) were downloaded from NCBI. The total 54,309,982 read count (∼108 million reads) were mapped to the rice *O. sativa japonica* cv. Nipponbare (*Os japonica*) reference genome information (version 7) from http://rice.plantbiology.msu.edu/ using SHORE (http://1001genomes.org/).

### SHORE & SHOREmap pipeline

The sequence SHORE analysis pipeline [26], [27], is a mapping and analysis pipeline for short DNA sequences retrieved from databases. The SHORE, short read analysis pipeline involves five different ‘OPTIONS’ for processing/analysis. A) SHORE preprocess: prepares index files, local repeat and GC content files from references. B) SHORE import: prepares short read data for processing. C) SHORE Mapflowcell: Short read mapping. D) SHORE merge: Merge and filter of alignment files and E) SHORE consensus: creates consensus sequence from alignment and computes homozygous SNP file with other results. It is designed for projects whose analysis strategy involves mapping of reads to a reference sequence. We used Burrows-Wheeler Aligner (BWA) to align short reads to the rice reference genome (MSU v7) with option -n4 -g3 -c12 and a default of 5 bases Cutoff for base masking using Sanger calibrated qualities, and Cutoff for base masking using chastity values [26], [28]. The Chastity value was introduced by Illumina's GA Pipeline and is used to differentiate between clusters, which are interfered by other clusters and those that are not. It is defined as the highest base intensity of a sequenced base, divided by the sum of the highest plus the second highest intensity of the specific base. Therefore the Chastity score can reach values from 0.5 to 1, or in percent from 50 to 100. The prediction of valid SNP calls have been made on the basis of SHORE statistics explained in http://sourceforge.net/apps/mediawiki/shore/index.php?title=Shore_consensus. All position SNP calls follow a concordance of ≥80% and support of at least three non-repetitive reads [26].

SHORE enables more than 50% of the overlapping reads with weighted and gapped alignments. We used the results of the alignments for discovery of putative SNPs, where each variation supported be at least 3 reads was regarded positive so as to eliminate false positive calls. SHORE calculates a quality score based on information from several features related to the quality of sequence reads and the alignment. The alignment results were subjected to quality filtering, and a cutoff of more than 25 out of 40 quality score was selected and used for functional SNP analysis. SHORE analysis includes heterozygous SNP calls (decision tree approach) for all positions with at least 25% of the bases different to the majority call. It has been reported that residual heterozygosity is present in the original individual used in the IRGSP sequencing, and a small subset of allelic sites within individuals have been identified [29] but due to statistical sampling and sequencing bias the accuracy of these SNPs is affected by heterozygous SNPs as well when sequencing heterozygous samples. Since these SNPs are not very significant for marker development, we did not follow this analysis further using these calls, and the final SNPs on annotated genic and non genic regions have been evaluated only with real homozygous SNPs [21], [30]. This quality SNP data was used for functional annotation with help of the SHOREmap pipeline and analyzed further by a suite of Linux scripts [27].

### SNP analysis on rice gene families and TF database

The rice genes with annotated SNPs were grouped in gene families according to their gene locus IDs in the MSU database (http://rice.plantbiology.msu.edu) and extracted with functional SNP annotation.

We could retrieve 2,726 gene loci from 32 rice gene families as described in the MSU rice genome database http://rice.plantbiology.msu.edu/annotation_community_families.shtml. In addition, SNPs were identified in transcription factors derived from the rice transcription factor database (DRTF) that currently contains 2,384 putative transcription factors (TF) gene models in *O. sativa spp. japonica*, distributed in 63 families [29], [31].

### Visualization of whole genome SNPs

The visualization of genome-wide SNPs was done with the CVit tool [32]. The SNP types were categorized based on the GFF (General Feature file) from the MSU rice genome annotation (version 7) and SNPs identified between Guangluai-4 and Nipponbare of the two sequenced genomes *Oryza sativa* (*Os*) *indica* and *Os japonica* respectively using SHOREmap [27].

The results of SHORE genome-wide base-by-base analysis of two reference rice genomes were used to develop the SNP2GENE database. We used MySQL as the underlying database to create the table structure, store the results and to allow for efficient searches. The front-end web interface to the database was written in php. We also developed a custom php module for visualization of a selected individual gene. The web interface to the SNP2GENE database is available at https://plantstress-pereira.uark.edu/oryza2.

## Results and Discussion

### Genome-wide natural polymorphisms

The NGS genome sequence of rice genotypes *O. sativa* ssp. *indica* cv. Guangluai-4 (G4) (*Os indica*) was analyzed with the objective to identify synonymous-SNP (sSNP) and non-synonymous-SNP (nsSNP) variation within genes. The complete genome sequence of rice *Os japonica* 12 pseudomolecules including the Syngenta pseudomolecules were used for SNP survey with *Os indica* next generation sequencing reads available at NCBI (Table 1). The analysis revealed more than 65% of the total genes with genic variation. A total of 207,659 SNPs were found on Chromosome 1, which contributes to the highest number in comparison to other chromosomes, followed by 174,855 SNPs in Chromosome 2 and 160,882 in chromosome 3, proportional to their size, as also seen for the other chromosomes (Table 2). The lowest rate of SNPs were observed in chromosome 9 (109797) followed by chromosome 12 (114308), which has been reported to contain a high number of disease resistance genes with recent gene duplications [33] (Table S1). We further grouped these SNPs into different types of polymorphisms, categorized as intergenic/noncoding, 3′-region, 5′-region, CDS, synonymous and non-synonymous and SNP2GENE database develop for this to retrieve the genome-wide information. In this analyses that covers 63% of genome between *japonica* Nipponbare and *indica* Guangluai-4, we found 40,761 gene loci having polymorphisms. Further analysis of these genes reveals 75,262 synonymous and 119,836 non-synonymous changes that may have functional significance. We found 22,686 SNPs at the three-prime (3′) UTR region and 23,242 five-prime (5′) UTR regions, and functional reading frame changes through 3,886 inducing STOP-codon (isSNP) and 729 preventing STOP-codon (psSNP) variation.

**Table 1:** Dataset and statistics of genotypes used in this study.

Sl.No.	Genome	Sub-species	Cultivar	covered	SNP	SNP/kb	data used
1	*Orzya sativa*	*Japonica*	Nipponbare	100%	Ref		MSU
2	*Orzya sativa*	*Indica*	Guangluai-4	63%	1674360	6.9	NCBI

**Table 2:** Distribution of SNPs and InDels on rice chromosomes.

		InDel Polymorphisms		SNP Polymorphisms					
Chr. No.	Total SNPs	Insertions	Deletions	Intergenic/noncoding	3′ UTR	5′ UTR	CDS	Synonymous	Non-synonymous
1	207659	9689	11551	37325	3315	3488	22401	9024	13377
2	174855	8136	9504	30976	2654	2598	18779	7217	11562
3	160882	7727	8897	28632	2524	2583	16287	6222	10065
4	125653	5513	6447	23141	1732	2016	16142	6302	9840
5	124752	5852	6579	20269	1791	1800	13617	5302	8315
6	138429	5939	7031	24814	1611	1572	16234	6222	10012
7	139709	6048	7006	23905	1996	1841	16690	6492	10198
8	120056	5223	6106	20683	1593	1726	14161	5432	8729
9	109797	4761	5482	20000	1214	1363	12955	4882	8073
10	123407	5039	5875	20766	1434	1388	15048	5723	9325
11	134853	5637	6202	24649	1421	1431	18649	7043	11606
12	114308	4730	5396	19351	1401	1436	14135	5401	8734
**Total**	1674360	80146	92655	294511	22686	23242	195098	75262	119836

### Gene based polymorphisms

**Figure 1:**
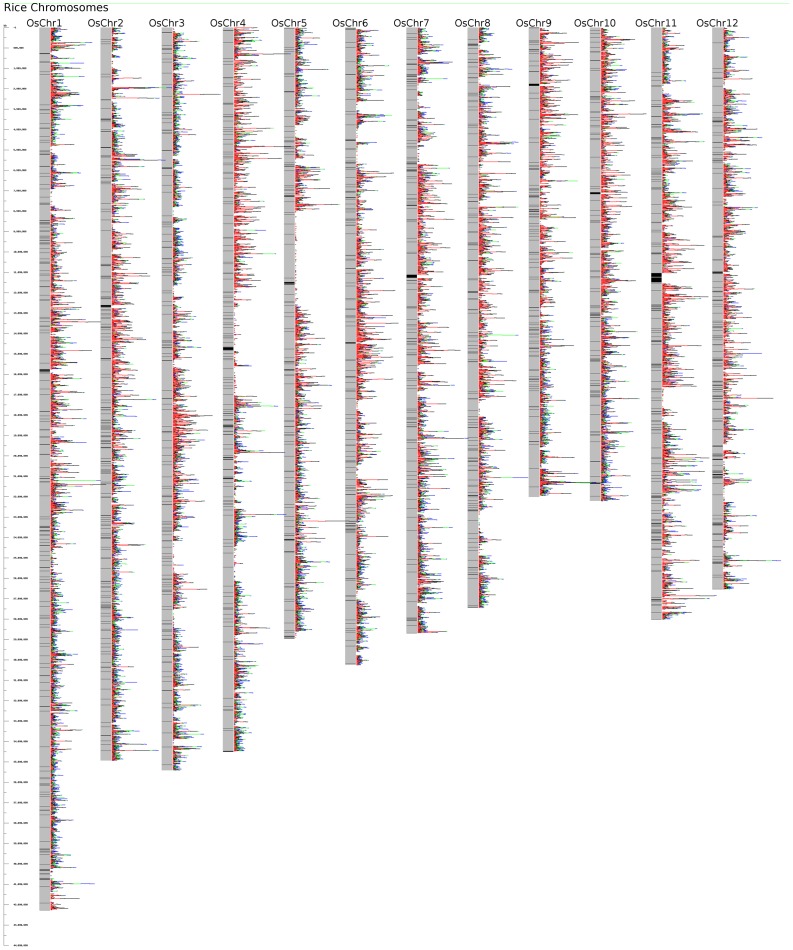
Distribution of gene based SNPs on 12 rice chromosomes, 55801 loci and 240914 positions. The SNPs glyph shows intergenic region; black, locus; gray, on chromosomes and range bar (left side of chromosomes) Syn-SNPs, black; Nonsyn-SNPs, red; threeprimeUTR-SNPs, blue; fiveprimeUTR-SNPs, green color.

We retrieved the total SNP information of the two rice genotypes based on 55,801 loci with 240,914 SNPs, comprising non-synonymous, synonymous, 5′-UTRs and 3′-UTRs on 12 rice chromosomes (Figure 1, Table S2 and Table S3). The functional analyses of the 55,801-gene annotation available (http//:www.plantbiology.msu.edu/) reveals 40,764 loci contribute to SNPs between these two genomes (Table S1). Since not all SNPs are important for functional analysis, the study was extended towards functional annotation of these SNPs and their effect on amino acid changes described in terms of synonymous and non-synonymous changes. These SNPs with putative functional changes were next used for gene family analysis with classifications available from annotation communities (http://rice.plantbiology.msu.edu). The locus information for the gene families were extracted and SNPs analyzed in the 2726 genes belonging to 32 different gene families and 104 sub-family groups curated by the rice community with functional and/or structural annotation submitted to the Rice Genome Annotation Project (http://rice.plantbiology.msu.edu/annotation_community_families.shtml). Our analysis shows 924 loci comprising10 different gene families are conserved, whereas other gene families have at least one variation in the family (Table 3). In total there are 239 SNP, comprising 21 synonymous and 31 non-synonymous SNPs in 2726 gene members. The largest gene family of proteins, the F-box has 10 families and 686 members that are completely conserved between these two rice sub-species, and have been reported to have conserved domains [34]. In contrast to that, 12 gene families possess synonymous and non-synonymous SNPs that have been reported to have important functions in plants.

**Table 3:** Distribution and types of SNPs in rice gene families.

Sl. Nos	Rice Gene Families	No. of families	No. of members	SNPs	Syn	Nonsyn
1	Ascorbate Peroxidase (APx) Family	1	8	19	2	0
2	ATP-dependent metalloprotease family	1	9	9	3	1
3	Bric-A-Brac/Tramtrack/Broad Complex (BTB) Proteins	18	145	4	2	1
4	C_2_H_2_ zinc finger proteins	1	179	3	1	2
5	Calmodulin and calmodulin-related calcium sensor proteins	2	37	0	0	0
6	Cellulose Synthase Gene Family	2	45	0	0	0
7	Conserved peptide uORF-containing transcripts	1	39	0	0	0
8	Core Replication Machinery Proteins	1	58	0	0	0
9	Cysteine Rice Peptides	28	532	6	1	5
10	Early Auxin-responsive Aux/IAA Gene Family	1	31	3	0	0
11	Early Auxin-responsive GH3 Gene Family		14	0	0	0
12	Endo-Beta-Mannanases	1	9	5	0	1
13	F-Box Proteins	10	686	0	0	0
14	Glutaredoxin family	1	28	0	0	0
15	Glycosyl Hydrolase Family 1 Beta-Glucosidases	1	38	8	1	2
16	HKT transporters	1	7	8	1	1
17	Indole-3-acetic-acid synthetase family	1	13	0	0	0
18	LIM domain proteins	1	6	2	0	0
19	Lipocalin Gene Family	2	3	6	2	0
20	MADS-box family	1	74	71	3	9
21	Mitogen-Activated Protein Kinases (MAPKs) and MAPK kinases (MAPKKs)	2	23	19	0	3
22	Phosphatidylethanolamine Binding Protein (PEBP) Gene Family	3	19	0	0	0
23	Protein Disulfide Isomerase Superfamily	3	23	22	0	1
24	RCI2 Homologs	1	11	0	0	0
25	Rice Kinase Interactome	1	149	8	2	1
26	SBP-Box Proteins	1	19	4	0	0
27	Serine Proteases	13	222	15	2	2
28	Small auxin-up gene family	1	58	0	0	0
29	Topoisomerase 6 homologs	1	4	17	1	1
30	Type-A Response Regulators	1	10	2	0	0
31	Wall-associated Kinases	1	144	1	0	1
32	WRKY Gene Superfamily	1	83	7	0	0

Amongst the gene families of interest are the MADS-box family (71 SNPs) that functions in flower and fruit development [35], the protein disulfide isomerase superfamily (22 SNPs) controls diverse metabolic functions including disulfide bond formation and isomerisation during protein folding [36], [37], ascorbate peroxidase (APx) family (19 SNPs) for development and response to environmental cues [38], [39], mitogen-activated protein kinases (MAPKs) and MAPK kinases (MAPKKs) with 19 SNPs that play an important role in response to pathogens and disease resistance cascades, topoisomerase 6 homologs (17 SNPs) involved in overexpression of stress tolerance genes [37]and serine proteases (15 SNPs) known for proteolytic enzymes associated with several essential physiological pathways [40]. The other 15 gene families have single digit SNPs and the rest 11 are conserved (Table 3). We grouped all identified genome wide SNPs in range of SNPs to discover the highest and lowest ranging SNP loci (Table 4 and Table S4).

**Table 4:** Number of genes in the given range of SNPs.

SNP range	Number of genes
1–5	15124
6–10	9070
11–15	5841
16–20	3352
21–25	2105
26–30	1067
31–35	955
36–40	670
41–45	519
46–50	882
51–55	250
56–60	206
61–65	208
66–70	154
71–75	99
76–80	86
81–85	59
86–90	67
91–95	44
96–100	32
>100	194

### Synonymous polymorphisms and non-synonymous polymorphisms

The importance of 1.6 million SNPs depends upon their position and effect on gene functions, although the non-functional variation is also important as markers in the genome. In recent years many of the gene based SNPs have been reported for their role in controlling characters like grain filling, plant height, grain weight, amylose content and structure of grain in rice and maize [17], [41], [42]. However, it has also been found that nsSNPs and sSNPs shared similar likelihood and size of effect in association to disease in humans [22].

**Figure 2:**
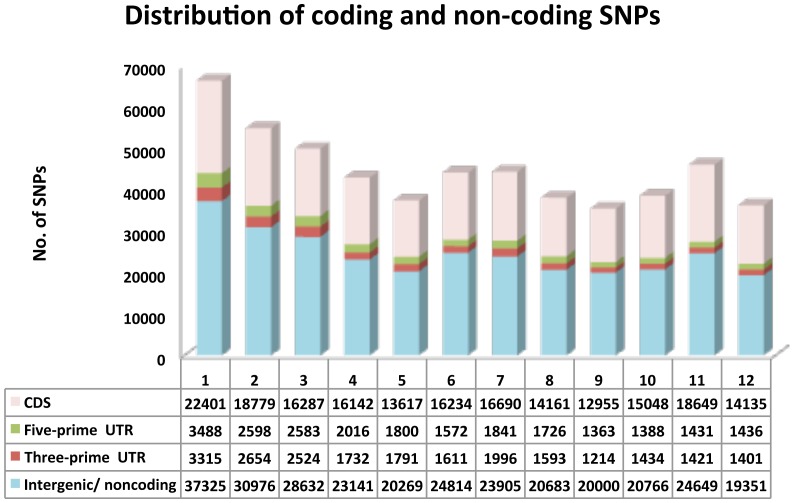
Distributions of SNPs on coding and non-coding region of rice 1–12 chromosomes. Chromosomes show CDS, Five-prime UTRs, Three-prime UTRs and intergenic non-coding SNPs from chromosome 1–12.

We found an interesting feature of SNP effect in our analysis, there were 195,446 SNPs on CDS (75,262 synonymous, 119,836 non-synonymous including 3,866 STOP codons) contributing 11% of the total SNPs effects related to change in proteins in the genomes (Figure 2), which is a significant contribution to putative phenotypic effects between these two subspecies. The highest number of non-synonymous SNP effects was found on Chr1 (13,377) followed by Chr2 (11,562). We found 3,886 SNPs which induced STOP-codons in the reading frames, and 729 SNPs preventing STOP-codon variation in the genome (Table S5 and Table S6). The highest number of induced STOP-codons was observed in Chr2 followed by Chr11 and minimum in Chr5.

**Figure 3:**
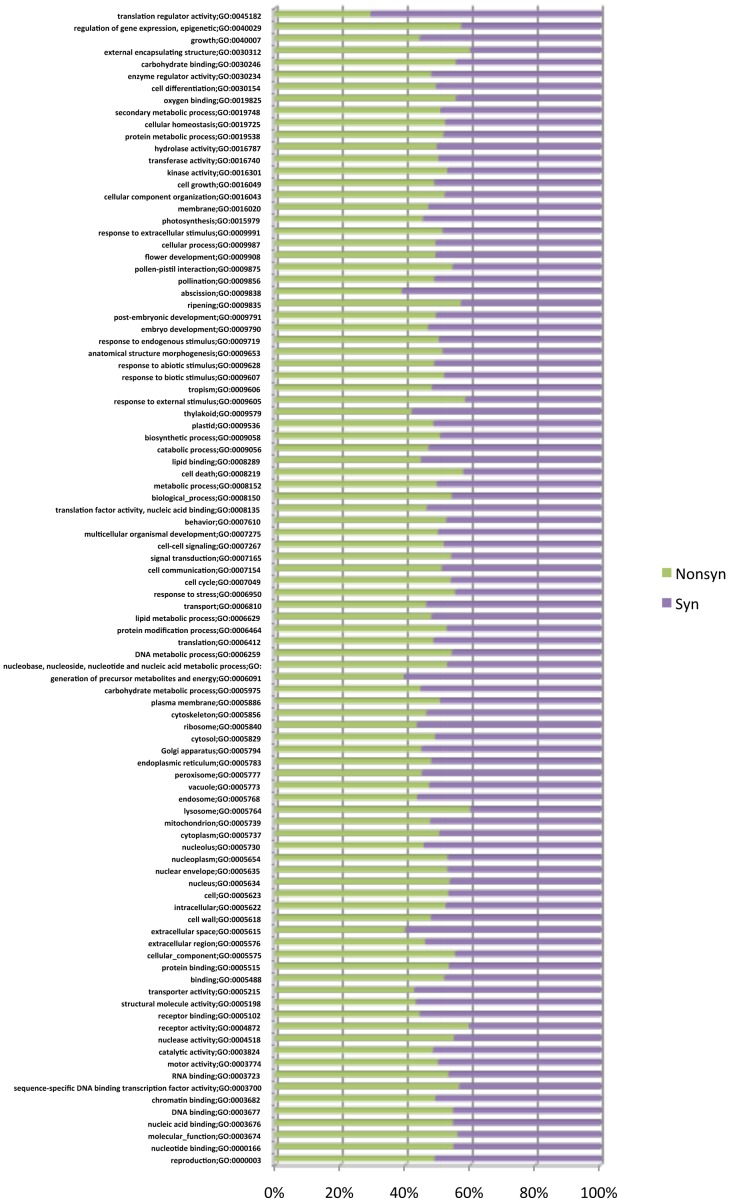
Analysis of all rice SNPs in genes with respect to their GO-terms descriptions.

The analysis was extended to identify the Ka/Ks ratios from the identified synonymous and non-synonymous changes for the kinase gene family, as an example to identify potential signatures of their biological functions, phenotypic effects, or expected evolutionary history. The family has potential roles in many traits such as pathogen defense, response to environment and almost all essential cellular functions. The predicted 1467 rice kinase genes were downloaded from http://phylomics.ucdavis.edu/kinase/. The IDs of these kinases were searched in the whole genome SNP analysis database, and 1313 genes showing SNPs were listed as kinase SNPs. We could find 839 kinases falling in the Ka/Ks (*Ka*  =  nonsynonymous substitutions number, and *Ks*  =  synonymous substitutions number) variation range from low (0.06) to high (12.0). There are 538 kinase genes that show more than 1.0 Ka/Ks value, with a potential signature for positive selection in evolution and a functional role in plants. These receptor kinases show more variability than other proteins signifying a selective role for the polymorphisms [43], [44] (Table S7). Similarly some gene ontology biological processes (Figure 3) such as ‘regulation of gene expression’, ‘response to external stimulus’, ‘receptor activity’ show a higher level of non-synonymous changes (55–60%), signifying a positive selection for variation. Induced mutations have been reported to create a large proportion of non-sense mutations involving the introduction of novel stop-codons, and any individual mutations are therefore more likely to have a phenotypic effect, and partly explain the high mutation frequency achieved in rice [45], [46]. The total 1.6 million SNPs were analyzed for the type of SNPs they carried, since this could affect the biological processes to a major extent.

### High SNP genes – hotspots for variation in the rice genome

Rice has undergone significant phenotypic changes during domestication in different traits like grain size, color, shattering, seed dormancy and tillering, and geneticists have used quantitative trait locus (QTL) mapping to localize the major causative genes responsible for these traits, yielding many trait-related genes in cultivated rice [14]. These phenotypic variations are due to natural genetic variation, which is high in number as seen in the variation between the two subspecies. However, the distributions of these natural variations are not uniform throughout the genomes. It has been reported that transposon induced points (TIP) have generated approximately 14% of the genomic DNA sequence differences between subspecies *indica* and *japonica* and 10% of TIPs have been found in expressed gene regions, which represent important genetic variation [47]. These regions have genic and intergenic regions and were not distributed homogeneously throughout the genic regions.

Our analysis of SNPs based on genic regions revealed that 40,761 genes carry 535,537 SNPs. The variation was as high as 33 fold in LOC_Os04g10740, LOC_Os04g10700, LOC_Os04g03100, LOC_Os12g43630 as compared to the average of 4.5 SNP/kb within the genic region. In fact, the genic region possesses 32% of the total genome SNPs and the top 33% (13,500) of SNP carrying genes contain 72% (387,816) SNPs from the genic region. We next focused on 194 hotspot SNP genes that carried>100 SNPs/gene each, and which contribute to 5% (26,080) of genic SNPs (Table S2).

### Variation in stress responsive transcription factors between sub-species

**Figure 4:**
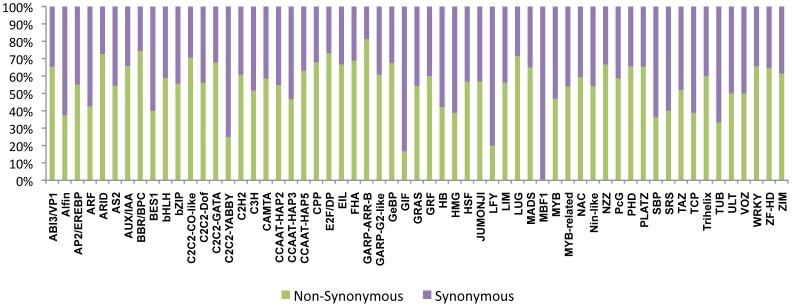
Synonymous and non-synonymous SNPs in transcription factor gene families. Variation in 2,384 putative transcription factors (TF) gene models in *O. sativa* spp. *japonica*, distributed in 63 families transcription factors have been predicted by DRTF.

Understanding the molecular basis of natural phenotypic diversity is a major challenge in modern genetics and knowing how individual genetic polymorphisms combine to produce phenotypic change could strengthen evolutionary theory and advance applications in crop improvement [48]. Transcription factor (TF) proteins, which form gene regulatory networks (GRNs) to act in cooperative or competitive partnerships to regulate gene expression, are key components of these unique regulatory programs [49]. Therefore, TFs are key players in affecting the expression of genes and leading to qualitative phenotypic changes. The interaction between TFs and cis-regulatory DNA sequences control gene expression, constituting the essential linkages of regulatory networks. Sequence variations in TFs have been reported to control and cause phenotypic shifts in trait expression. Based on the fact that genetic interactions between transcriptions factors are a major source of phenotypic diversity within the species [48], we explored the TF locus information and analyzed the natural variation to understand their adaptive evolution. The analyzed regulatory SNPs that are located in the 3′ and 5′ flanking regions of different rice genes that might influence the activity of the transcriptional regulatory region, which comprise the cis-regulatory regions of the genes [50]. The predicted transcription factors of *Os japonica* in the DRTF currently comprise 2,384 putative transcription factor (TF) gene models, distributed in 63 families [31]. These TF genes were studied for their functional SNPs, as shown in Figure 4 and Table 5. Differences in gene expression may play a major role in speciation and phenotypic diversity.

**Table 5:** Distribution of polymorphism types in rice transcription factors.

TF family	Gene Number*	Total SNP	Non-Synonymous	Synonymous
ABI3/VP1	57	357	51	27
Alfin	13	103	3	5
AP2/EREBP	182	785	115	94
ARF	41	443	55	74
ARID	7	70	8	3
AS2	39	161	37	31
AUX/IAA	46	237	23	12
BBR/BPC	7	70	26	9
BES1	6	27	2	3
bHLH	184	1491	172	120
bZIP	109	1046	98	78
C2C2-CO-like	54	416	48	20
C2C2-Dof	36	166	23	18
C2C2-GATA	23	85	21	10
C2C2-YABBY	12	65	1	3
C2H2	113	984	161	104
C3H	90	677	103	96
CAMTA	8	159	24	17
CCAAT-Dr1	1	14	0	0
CCAAT-HAP2	20	224	17	14
CCAAT-HAP3	17	56	7	8
CCAAT-HAP5	18	165	46	27
CPP	16	109	17	8
E2F/DP	9	130	11	4
EIL	12	42	12	6
FHA	19	245	42	19
GARP-ARR-B	10	74	13	3
GARP-G2-like	56	534	51	33
GeBP	15	97	27	13
GIF	3	42	1	5
GRAS	58	392	89	75
GRF	18	58	12	8
HB	103	843	83	114
HMG	19	100	7	11
HRT-like	1	0	0	0
HSF	36	312	29	22
JUMONJI	17	282	37	28
LFY	1	13	1	4
LIM	13	155	45	35
LUG	11	108	15	6
MADS	83	1020	74	40
MBF1	3	7	0	2
MYB	138	1286	168	190
MYB-related	84	913	115	98
NAC	149	953	200	137
Nin-like	14	139	26	22
NZZ	1	4	2	1
PBF-2-like Whirly	1	6	0	0
PcG	34	538	78	55
PHD	79	1236	249	130
PLATZ	20	170	17	9
S1Fa-like	4	10	0	0
SBP	28	184	8	14
SRS	6	19	2	3
TAZ	10	131	26	24
TCP	24	82	7	11
Trihelix	23	123	21	14
TUB	21	158	12	24
ULT	2	32	2	2
VOZ	2	34	2	2
WRKY	113	1067	202	106
ZF-HD	15	113	31	17
ZIM	29	200	16	10

*The number of transcription factors have been predicted by DRTF (Gao et al., 2006).

## Conclusions

Asian rice *Oryza sativa* is a cultivated, inbreeding species that feeds over half of the world's population. Genome-wide gene based analysis of sequence polymorphisms between the rice two major cultivated rice subspecies, *O. sativa* ssp *japonica* cv. Nipponbare and *O. sativa* ssp *indica* cv. Guangluai-4, revealed the distribution of SNPs and Insertion/Deletions at the gene level. The sequence polymorphisms commonly occur in both coding and non-coding regions and have significant differences in distribution between different gene families. These variations often affect differences in gene structure between the *indica* and *japonica* subspecies, and may contribute to phenotypic adaptation differences.

## Supporting Information

Table S1 **SNP distributions of all rice genes of 12 chromosomes.**
(XLSX)

Table S2 **Distributions of 3′ UTR SNPs in rice Chromosome 1-12.**
(XLSX)

Table S3 **Distributions of 5′ UTR SNPs in rice Chromosome 1-12.**
(XLSX)

Table S4 **Hotspot genes containing 100 SNP and more in the locus.**
(XLSX)

Table S5 **Distributions of ‘Induced Stop’ coding SNPs in rice Chromosomes 1-12.**
(XLSX)

Table S6 **Distribution of Stop codon preventing SNPs in rice Chromosome 1.**
(XLSX)

Table S7 **Distribution of Rice Kinase genes Ka/Ks ratio distribution.**
(XLSX)
